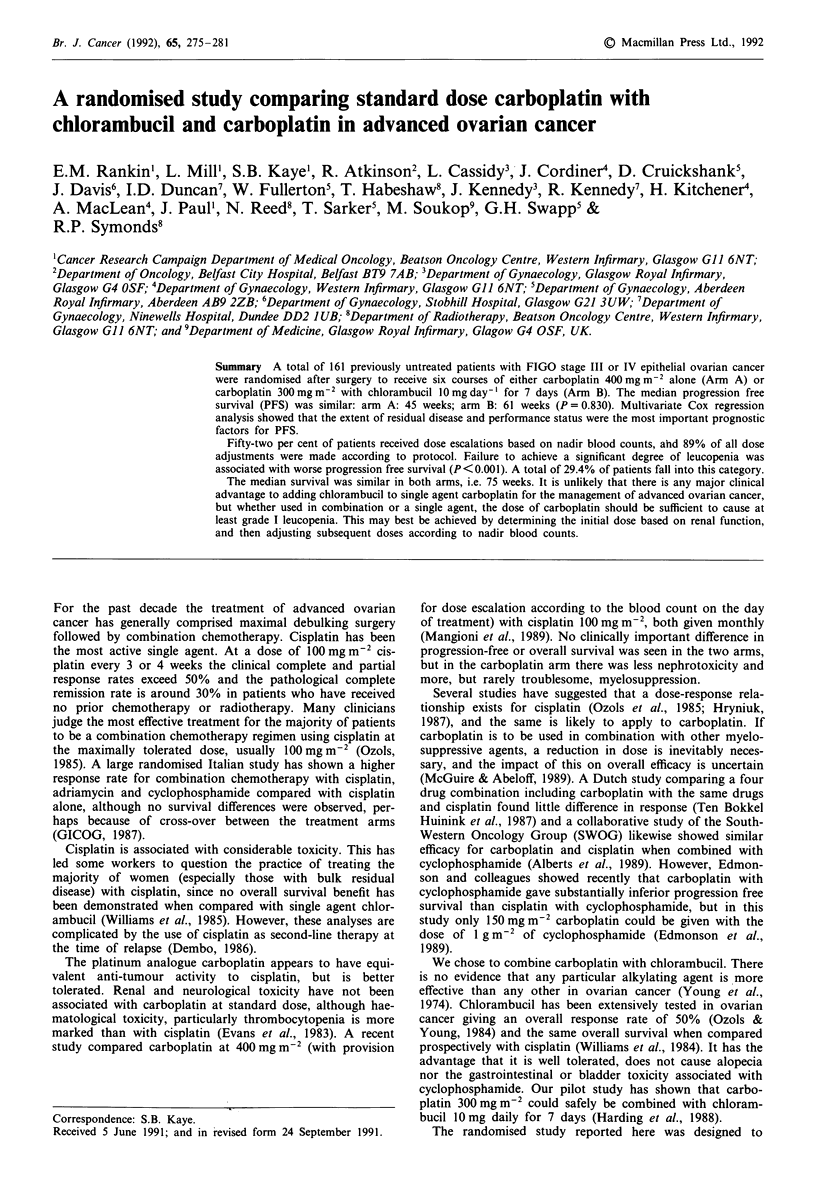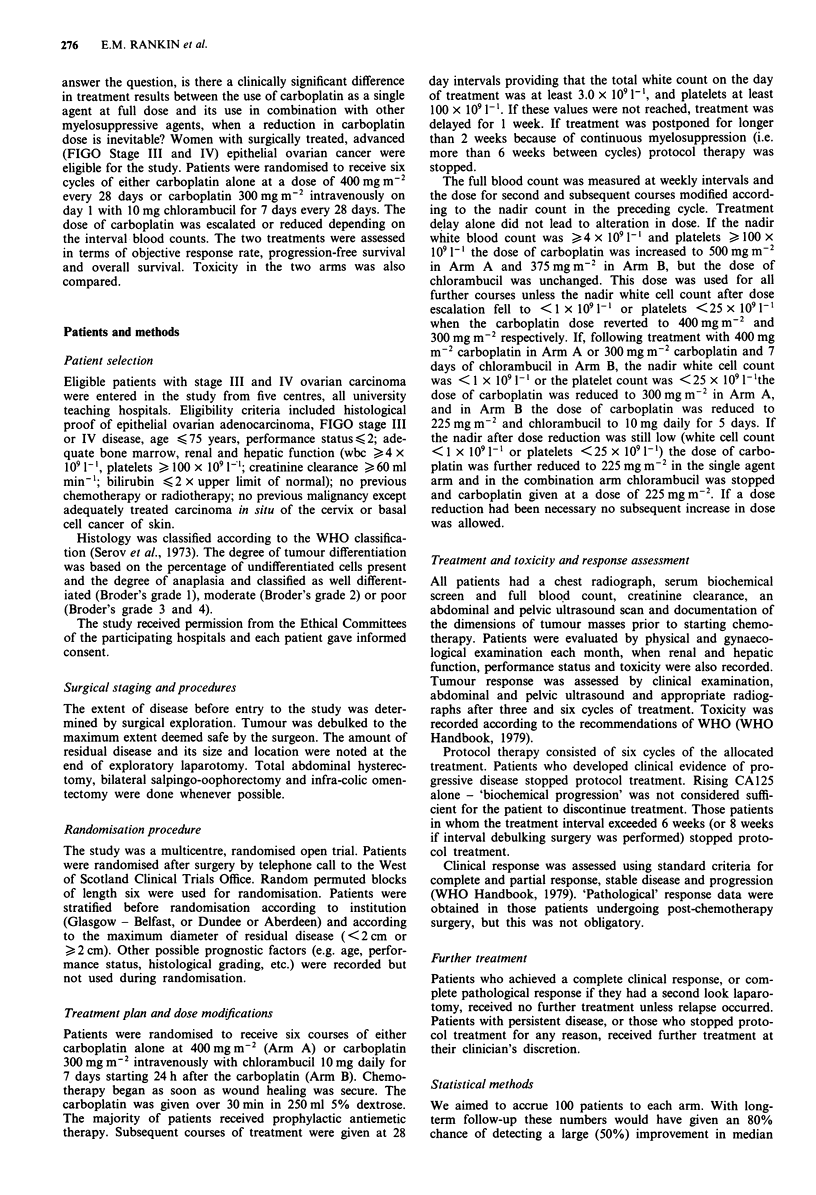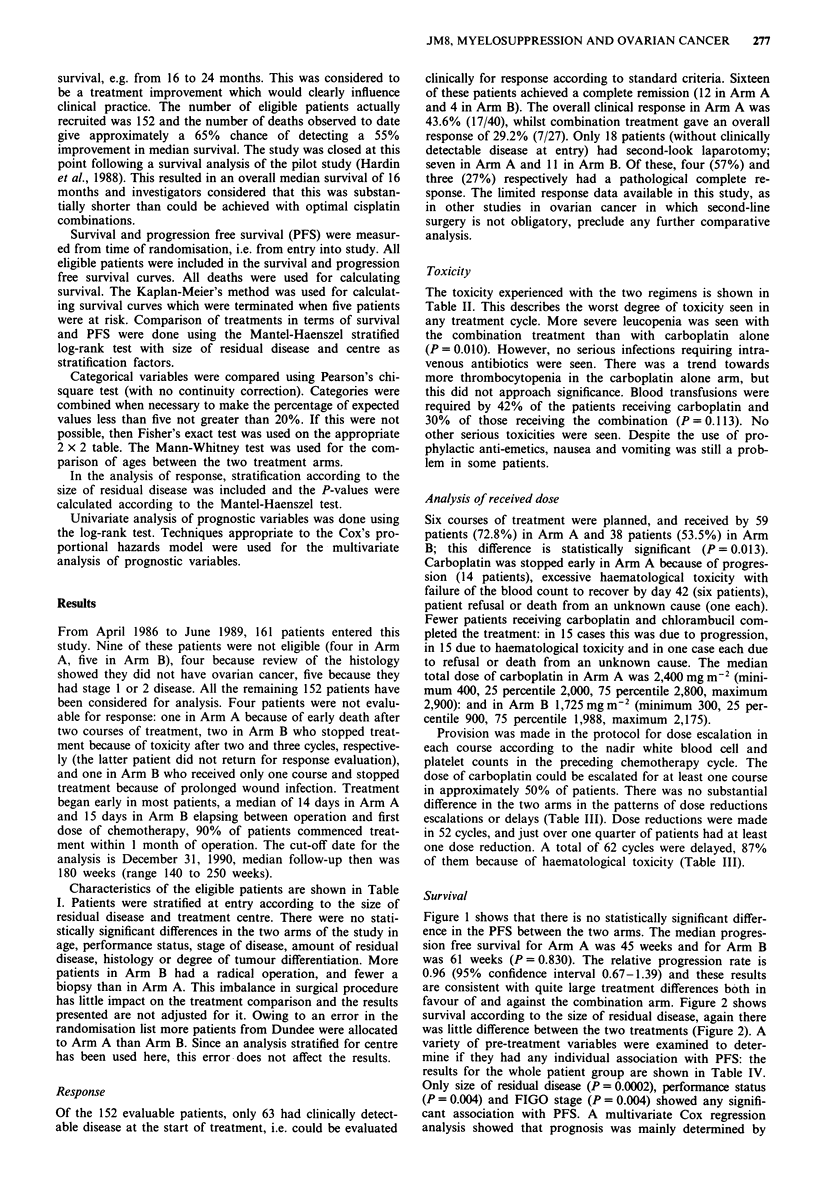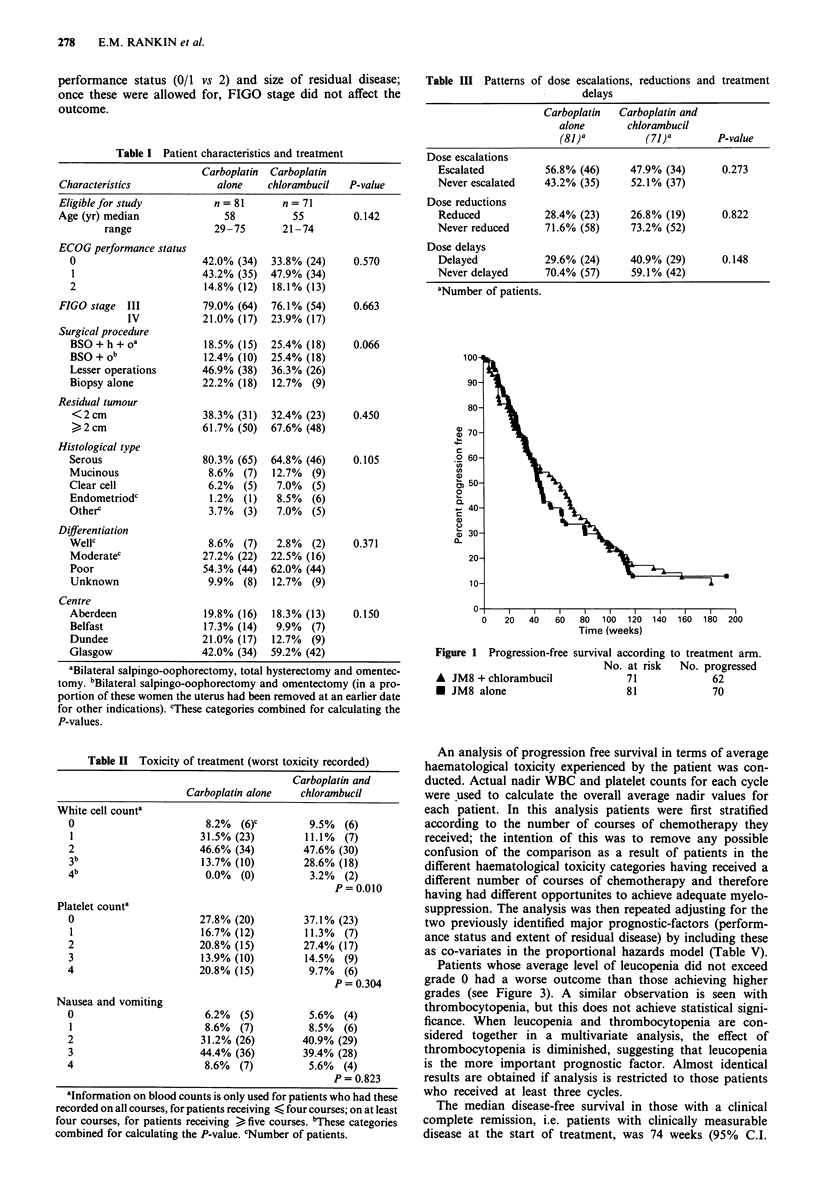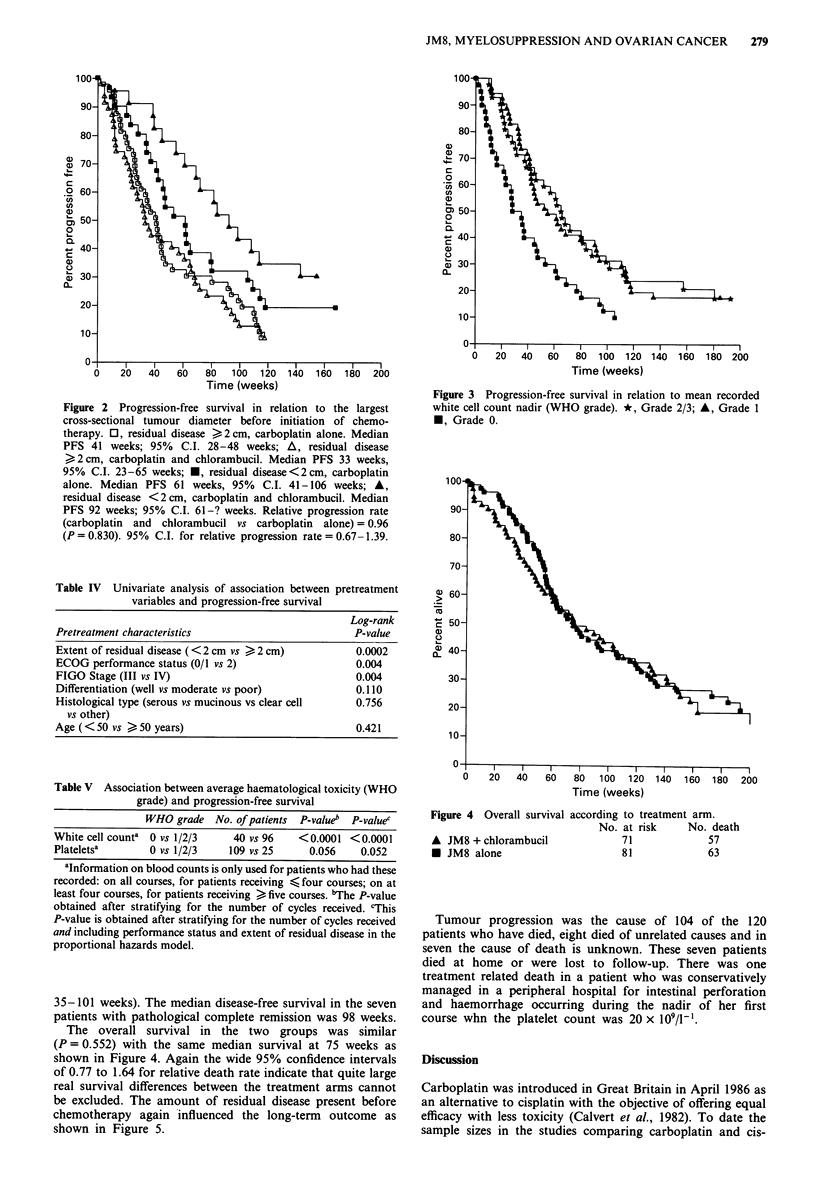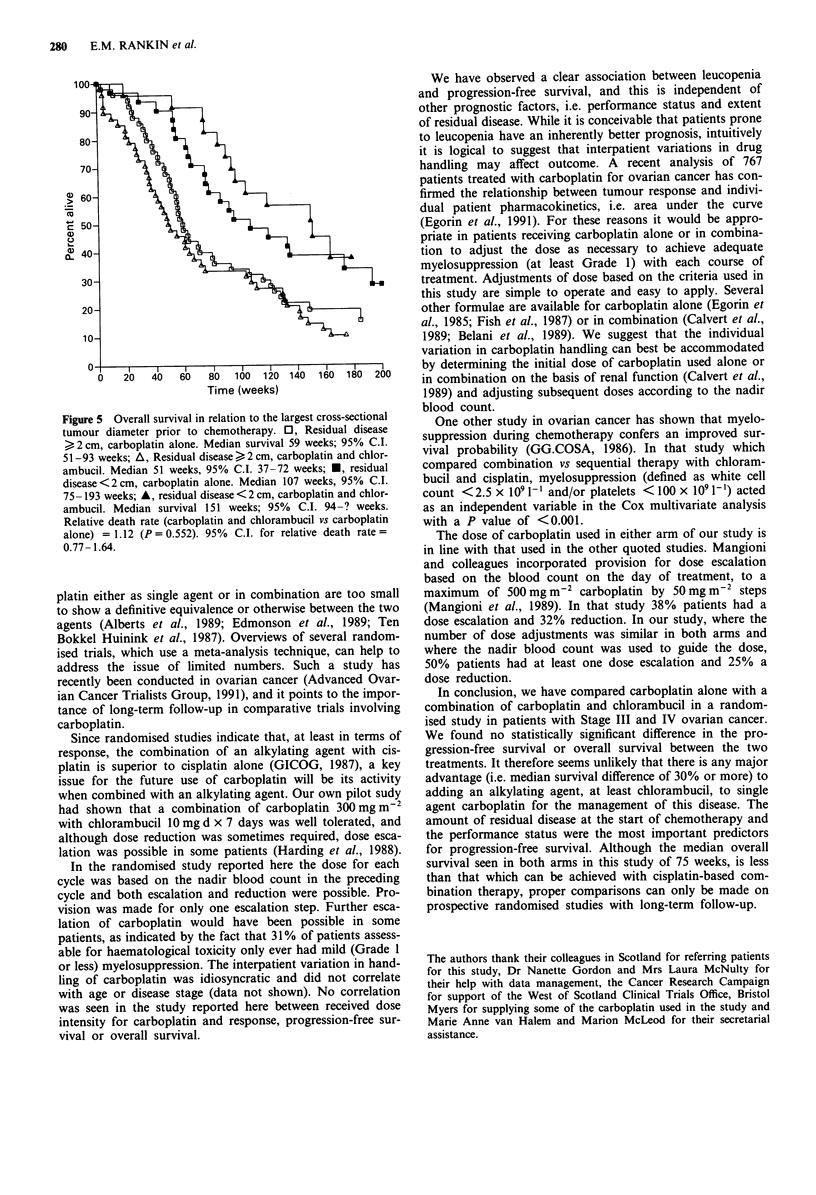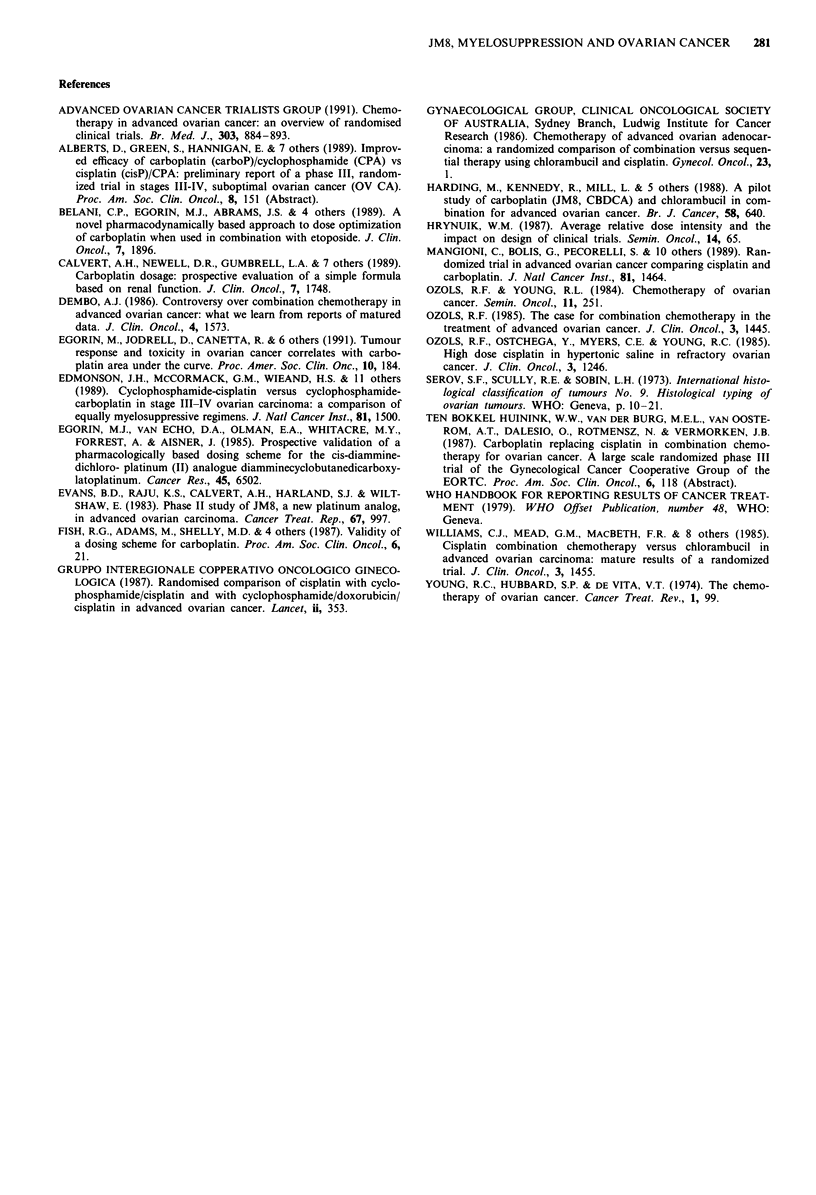# A randomised study comparing standard dose carboplatin with chlorambucil and carboplatin in advanced ovarian cancer.

**DOI:** 10.1038/bjc.1992.55

**Published:** 1992-02

**Authors:** E. M. Rankin, L. Mill, S. B. Kaye, R. Atkinson, L. Cassidy, J. Cordiner, D. Cruickshank, J. Davis, I. D. Duncan, W. Fullerton

**Affiliations:** Cancer Research Campaign Department of Medical Oncology, Beatson Oncology Centre, Western Infirmary, Glasgow, UK.

## Abstract

A total of 161 previously untreated patients with FIGO stage III or IV epithelial ovarian cancer were randomised after surgery to receive six courses of either carboplatin 400 mg m-2 alone (Arm A) or carboplatin 300 mg m-2 with chlorambucil 10 mg day-1 for 7 days (Arm B). The median progression free survival (PFS) was similar: arm A: 45 weeks; arm B: 61 weeks (P = 0.830). Multivariate Cox regression analysis showed that the extent of residual disease and performance status were the most important prognostic factors for PFS. Fifty-two per cent of patients received dose escalations based on nadir blood counts, and 89% of all dose adjustments were made according to protocol. Failure to achieve a significant degree of leucopenia was associated with worse progression free survival (P less than 0.001). A total of 29.4% of patients fall into this category. The median survival was similar in both arms, i.e. 75 weeks. It is unlikely that there is any major clinical advantage to adding chlorambucil to single agent carboplatin for the management of advanced ovarian cancer, but whether used in combination or a single agent, the dose of carboplatin should be sufficient to cause at least grade I leucopenia. This may best be achieved by determining the initial dose based on renal function, and then adjusting subsequent doses according to nadir blood counts.


Br. J. Cancer (1992), 65, 275–281 © Macmillan Press Ltd., 1992

### A randomised study comparing standard dose carboplatin with chlorambucil and carboplatin in advanced ovarian cancer

E.M. Rankin[1], L. Mill[1], S.B. Kaye[1], R. Atkinson[2], L. Cassidy[3], J. Cordiner[4], D. Cruickshank[5], J. Davis[6], I.D. Duncan[7], W. Fullerton[5], T. Habeshaw[8], J. Kennedy[3], R. Kennedy[7], H. Kitchener[4], A. MacLean[4], J. Paul[1], N. Reed[8], T. Sarker[5], M. Soukop[9], G.H. Swapp[5] & R.P. Symonds[8]

[1]*Cancer Research Campaign Department of Medical Oncology, Beatson Oncology Centre, Western Infirmary, Glasgow G11 6NT;* [2]*Department of Oncology, Belfast City Hospital, Belfast BT9 7AB;* [3]*Department of Gynaecology, Glasgow Royal Infirmary, Glasgow G4 0SF;* [4]*Department of Gynaecology, Western Infirmary, Glasgow G11 6NT;* [5]*Department of Gynaecology, Aberdeen Royal Infirmary, Aberdeen AB9 2ZB;* [6]*Department of Gynaecology, Stobhill Hospital, Glasgow G21 3UW;* [7]*Department of Gynaecology, Ninewells Hospital, Dundee DD2 1UB;* [8]*Department of Radiotherapy, Beatson Oncology Centre, Western Infirmary, Glasgow G11 6NT; and* [9]*Department of Medicine, Glasgow Royal Infirmary, Glagow G4 OSF, UK.*

**Summary** A total of 161 previously untreated patients with FIGO stage III or IV epithelial ovarian cancer were randomised after surgery to receive six courses of either carboplatin 400 mg m$^{-2}$ alone (Arm A) or carboplatin 300 mg m$^{-2}$ with chlorambucil 10 mg day$^{-1}$ for 7 days (Arm B). The median progression free survival (PFS) was similar: arm A: 45 weeks; arm B: 61 weeks ($P = 0.830$). Multivariate Cox regression analysis showed that the extent of residual disease and performance status were the most important prognostic factors for PFS.

Fifty-two per cent of patients received dose escalations based on nadir blood counts, and 89% of all dose adjustments were made according to protocol. Failure to achieve a significant degree of leucopenia was associated with worse progression free survival ($P < 0.001$). A total of 29.4% of patients fall into this category.

The median survival was similar in both arms, i.e. 75 weeks. It is unlikely that there is any major clinical advantage to adding chlorambucil to single agent carboplatin for the management of advanced ovarian cancer, but whether used in combination or a single agent, the dose of carboplatin should be sufficient to cause at least grade I leucopenia. This may best be achieved by determining the initial dose based on renal function, and then adjusting subsequent doses according to nadir blood counts.

For the past decade the treatment of advanced ovarian cancer has generally comprised maximal debulking surgery followed by combination chemotherapy. Cisplatin has been the most active single agent. At a dose of 100 mg m$^{-2}$ cisplatin every 3 or 4 weeks the clinical complete and partial response rates exceed 50% and the pathological complete remission rate is around 30% in patients who have received no prior chemotherapy or radiotherapy. Many clinicians judge the most effective treatment for the majority of patients to be a combination chemotherapy regimen using cisplatin at the maximally tolerated dose, usually 100 mg m$^{-2}$ (Ozols, 1985). A large randomised Italian study has shown a higher response rate for combination chemotherapy with cisplatin, adriamycin and cyclophosphamide compared with cisplatin alone, although no survival differences were observed, perhaps because of cross-over between the treatment arms (GICOG, 1987).

Cisplatin is associated with considerable toxicity. This has led some workers to question the practice of treating the majority of women (especially those with bulk residual disease) with cisplatin, since no overall survival benefit has been demonstrated when compared with single agent chlorambucil (Williams *et al.*, 1985). However, these analyses are complicated by the use of cisplatin as second-line therapy at the time of relapse (Dembo, 1986).

The platinum analogue carboplatin appears to have equivalent anti-tumour activity to cisplatin, but is better tolerated. Renal and neurological toxicity have not been associated with carboplatin at standard dose, although haematological toxicity, particularly thrombocytopenia is more marked than with cisplatin (Evans *et al.*, 1983). A recent study compared carboplatin at 400 mg m$^{-2}$ (with provision for dose escalation according to the blood count on the day of treatment) with cisplatin 100 mg m$^{-2}$, both given monthly (Mangioni *et al.*, 1989). No clinically important difference in progression-free or overall survival was seen in the two arms, but in the carboplatin arm there was less nephrotoxicity and more, but rarely troublesome, myelosuppression.

Several studies have suggested that a dose-response relationship exists for cisplatin (Ozols *et al.*, 1985; Hryniuk, 1987), and the same is likely to apply to carboplatin. If carboplatin is to be used in combination with other myelosuppressive agents, a reduction in dose is inevitably necessary, and the impact of this on overall efficacy is uncertain (McGuire & Abeloff, 1989). A Dutch study comparing a four drug combination including carboplatin with the same drugs and cisplatin found little difference in response (Ten Bokkel Huinink *et al.*, 1987) and a collaborative study of the South-Western Oncology Group (SWOG) likewise showed similar efficacy for carboplatin and cisplatin when combined with cyclophosphamide (Alberts *et al.*, 1989). However, Edmonson and colleagues showed recently that carboplatin with cyclophosphamide gave substantially inferior progression free survival than cisplatin with cyclophosphamide, but in this study only 150 mg m$^{-2}$ carboplatin could be given with the dose of 1 g m$^{-2}$ of cyclophosphamide (Edmonson *et al.*, 1989).

We chose to combine carboplatin with chlorambucil. There is no evidence that any particular alkylating agent is more effective than any other in ovarian cancer (Young *et al.*, 1974). Chlorambucil has been extensively tested in ovarian cancer giving an overall response rate of 50% (Ozols & Young, 1984) and the same overall survival when compared prospectively with cisplatin (Williams *et al.*, 1984). It has the advantage that it is well tolerated, does not cause alopecia nor the gastrointestinal or bladder toxicity associated with cyclophosphamide. Our pilot study has shown that carboplatin 300 mg m$^{-2}$ could safely be combined with chlorambucil 10 mg daily for 7 days (Harding *et al.*, 1988).

The randomised study reported here was designed to

Correspondence: S.B. Kaye.
Received 5 June 1991; and in revised form 24 September 1991.

answer the question, is there a clinically significant difference in treatment results between the use of carboplatin as a single agent at full dose and its use in combination with other myelosuppressive agents, when a reduction in carboplatin dose is inevitable? Women with surgically treated, advanced (FIGO Stage III and IV) epithelial ovarian cancer were eligible for the study. Patients were randomised to receive six cycles of either carboplatin alone at a dose of 400 mg $m^{-2}$ every 28 days or carboplatin 300 mg $m^{-2}$ intravenously on day 1 with 10 mg chlorambucil for 7 days every 28 days. The dose of carboplatin was escalated or reduced depending on the interval blood counts. The two treatments were assessed in terms of objective response rate, progression-free survival and overall survival. Toxicity in the two arms was also compared.

### Patients and methods

### *Patient selection*

Eligible patients with stage III and IV ovarian carcinoma were entered in the study from five centres, all university teaching hospitals. Eligibility criteria included histological proof of epithelial ovarian adenocarcinoma, FIGO stage III or IV disease, age $\leqslant$75 years, performance status $\leqslant$2; adequate bone marrow, renal and hepatic function (wbc $\geqslant 4 \times 10^9 l^{-1}$, platelets $\geqslant 100 \times 10^9 l^{-1}$; creatinine clearance $\geqslant$60 ml $min^{-1}$; bilirubin $\leqslant 2 \times$ upper limit of normal); no previous chemotherapy or radiotherapy; no previous malignancy except adequately treated carcinoma *in situ* of the cervix or basal cell cancer of skin.

Histology was classified according to the WHO classification (Serov *et al.*, 1973). The degree of tumour differentiation was based on the percentage of undifferentiated cells present and the degree of anaplasia and classified as well differentiated (Broder's grade 1), moderate (Broder's grade 2) or poor (Broder's grade 3 and 4).

The study received permission from the Ethical Committees of the participating hospitals and each patient gave informed consent.

### *Surgical staging and procedures*

The extent of disease before entry to the study was determined by surgical exploration. Tumour was debulked to the maximum extent deemed safe by the surgeon. The amount of residual disease and its size and location were noted at the end of exploratory laparotomy. Total abdominal hysterectomy, bilateral salpingo-oophorectomy and infra-colic omentectomy were done whenever possible.

### *Randomisation procedure*

The study was a multicentre, randomised open trial. Patients were randomised after surgery by telephone call to the West of Scotland Clinical Trials Office. Random permuted blocks of length six were used for randomisation. Patients were stratified before randomisation according to institution (Glasgow – Belfast, or Dundee or Aberdeen) and according to the maximum diameter of residual disease ($<$2 cm or $\geqslant$2 cm). Other possible prognostic factors (e.g. age, performance status, histological grading, etc.) were recorded but not used during randomisation.

### *Treatment plan and dose modifications*

Patients were randomised to receive six courses of either carboplatin alone at 400 mg $m^{-2}$ (Arm A) or carboplatin 300 mg $m^{-2}$ intravenously with chlorambucil 10 mg daily for 7 days starting 24 h after the carboplatin (Arm B). Chemotherapy began as soon as wound healing was secure. The carboplatin was given over 30 min in 250 ml 5% dextrose. The majority of patients received prophylactic antiemetic therapy. Subsequent courses of treatment were given at 28 day intervals providing that the total white count on the day of treatment was at least $3.0 \times 10^9 l^{-1}$, and platelets at least $100 \times 10^9 l^{-1}$. If these values were not reached, treatment was delayed for 1 week. If treatment was postponed for longer than 2 weeks because of continuous myelosuppression (i.e. more than 6 weeks between cycles) protocol therapy was stopped.

The full blood count was measured at weekly intervals and the dose for second and subsequent courses modified according to the nadir count in the preceding cycle. Treatment delay alone did not lead to alteration in dose. If the nadir white blood count was $\geqslant 4 \times 10^9 l^{-1}$ and platelets $\geqslant 100 \times 10^9 l^{-1}$ the dose of carboplatin was increased to 500 mg $m^{-2}$ in Arm A and 375 mg $m^{-2}$ in Arm B, but the dose of chlorambucil was unchanged. This dose was used for all further courses unless the nadir white cell count after dose escalation fell to $<1 \times 10^9 l^{-1}$ or platelets $<25 \times 10^9 l^{-1}$ when the carboplatin dose reverted to 400 mg $m^{-2}$ and 300 mg $m^{-2}$ respectively. If, following treatment with 400 mg $m^{-2}$ carboplatin in Arm A or 300 mg $m^{-2}$ carboplatin and 7 days of chlorambucil in Arm B, the nadir white cell count was $<1 \times 10^9 l^{-1}$ or the platelet count was $<25 \times 10^9 l^{-1}$the dose of carboplatin was reduced to 300 mg $m^{-2}$ in Arm A, and in Arm B the dose of carboplatin was reduced to 225 mg $m^{-2}$ and chlorambucil to 10 mg daily for 5 days. If the nadir after dose reduction was still low (white cell count $<1 \times 10^9 l^{-1}$ or platelets $<25 \times 10^9 l^{-1}$) the dose of carboplatin was further reduced to 225 mg $m^{-2}$ in the single agent arm and in the combination arm chlorambucil was stopped and carboplatin given at a dose of 225 mg $m^{-2}$. If a dose reduction had been necessary no subsequent increase in dose was allowed.

### *Treatment and toxicity and response assessment*

All patients had a chest radiograph, serum biochemical screen and full blood count, creatinine clearance, an abdominal and pelvic ultrasound scan and documentation of the dimensions of tumour masses prior to starting chemotherapy. Patients were evaluated by physical and gynaecological examination each month, when renal and hepatic function, performance status and toxicity were also recorded. Tumour response was assessed by clinical examination, abdominal and pelvic ultrasound and appropriate radiographs after three and six cycles of treatment. Toxicity was recorded according to the recommendations of WHO (WHO Handbook, 1979).

Protocol therapy consisted of six cycles of the allocated treatment. Patients who developed clinical evidence of progressive disease stopped protocol treatment. Rising CA125 alone – 'biochemical progression' was not considered sufficient for the patient to discontinue treatment. Those patients in whom the treatment interval exceeded 6 weeks (or 8 weeks if interval debulking surgery was performed) stopped protocol treatment.

Clinical response was assessed using standard criteria for complete and partial response, stable disease and progression (WHO Handbook, 1979). 'Pathological' response data were obtained in those patients undergoing post-chemotherapy surgery, but this was not obligatory.

### *Further treatment*

Patients who achieved a complete clinical response, or complete pathological response if they had a second look laparotomy, received no further treatment unless relapse occurred. Patients with persistent disease, or those who stopped protocol treatment for any reason, received further treatment at their clinician's discretion.

### *Statistical methods*

We aimed to accrue 100 patients to each arm. With long-term follow-up these numbers would have given an 80% chance of detecting a large (50%) improvement in median survival, e.g. from 16 to 24 months. This was considered to be a treatment improvement which would clearly influence clinical practice. The number of eligible patients actually recruited was 152 and the number of deaths observed to date give approximately a 65% chance of detecting a 55% improvement in median survival. The study was closed at this point following a survival analysis of the pilot study (Hardin *et al.*, 1988). This resulted in an overall median survival of 16 months and investigators considered that this was substantially shorter than could be achieved with optimal cisplatin combinations.

Survival and progression free survival (PFS) were measured from time of randomisation, i.e. from entry into study. All eligible patients were included in the survival and progression free survival curves. All deaths were used for calculating survival. The Kaplan-Meier's method was used for calculating survival curves which were terminated when five patients were at risk. Comparison of treatments in terms of survival and PFS were done using the Mantel-Haenszel stratified log-rank test with size of residual disease and centre as stratification factors.

Categorical variables were compared using Pearson's chi-square test (with no continuity correction). Categories were combined when necessary to make the percentage of expected values less than five not greater than 20%. If this were not possible, then Fisher's exact test was used on the appropriate $2 \times 2$ table. The Mann-Whitney test was used for the comparison of ages between the two treatment arms.

In the analysis of response, stratification according to the size of residual disease was included and the $P$-values were calculated according to the Mantel-Haenszel test.

Univariate analysis of prognostic variables was done using the log-rank test. Techniques appropriate to the Cox's proportional hazards model were used for the multivariate analysis of prognostic variables.

### Results

From April 1986 to June 1989, 161 patients entered this study. Nine of these patients were not eligible (four in Arm A, five in Arm B), four because review of the histology showed they did not have ovarian cancer, five because they had stage 1 or 2 disease. All the remaining 152 patients have been considered for analysis. Four patients were not evaluable for response: one in Arm A because of early death after two courses of treatment, two in Arm B who stopped treatment because of toxicity after two and three cycles, respectively (the latter patient did not return for response evaluation), and one in Arm B who received only one course and stopped treatment because of prolonged wound infection. Treatment began early in most patients, a median of 14 days in Arm A and 15 days in Arm B elapsing between operation and first dose of chemotherapy, 90% of patients commenced treatment within 1 month of operation. The cut-off date for the analysis is December 31, 1990, median follow-up then was 180 weeks (range 140 to 250 weeks).

Characteristics of the eligible patients are shown in Table I. Patients were stratified at entry according to the size of residual disease and treatment centre. There were no statistically significant differences in the two arms of the study in age, performance status, stage of disease, amount of residual disease, histology or degree of tumour differentiation. More patients in Arm B had a radical operation, and fewer a biopsy than in Arm A. This imbalance in surgical procedure has little impact on the treatment comparison and the results presented are not adjusted for it. Owing to an error in the randomisation list more patients from Dundee were allocated to Arm A than Arm B. Since an analysis stratified for centre has been used here, this error does not affect the results.

### *Response*

Of the 152 evaluable patients, only 63 had clinically detectable disease at the start of treatment, i.e. could be evaluated clinically for response according to standard criteria. Sixteen of these patients achieved a complete remission (12 in Arm A and 4 in Arm B). The overall clinical response in Arm A was 43.6% (17/40), whilst combination treatment gave an overall response of 29.2% (7/27). Only 18 patients (without clinically detectable disease at entry) had second-look laparotomy; seven in Arm A and 11 in Arm B. Of these, four (57%) and three (27%) respectively had a pathological complete response. The limited response data available in this study, as in other studies in ovarian cancer in which second-line surgery is not obligatory, preclude any further comparative analysis.

### *Toxicity*

The toxicity experienced with the two regimens is shown in Table II. This describes the worst degree of toxicity seen in any treatment cycle. More severe leucopenia was seen with the combination treatment than with carboplatin alone ($P = 0.010$). However, no serious infections requiring intravenous antibiotics were seen. There was a trend towards more thrombocytopenia in the carboplatin alone arm, but this did not approach significance. Blood transfusions were required by 42% of the patients receiving carboplatin and 30% of those receiving the combination ($P = 0.113$). No other serious toxicities were seen. Despite the use of prophylactic anti-emetics, nausea and vomiting was still a problem in some patients.

### *Analysis of received dose*

Six courses of treatment were planned, and received by 59 patients (72.8%) in Arm A and 38 patients (53.5%) in Arm B; this difference is statistically significant ($P = 0.013$). Carboplatin was stopped early in Arm A because of progression (14 patients), excessive haematological toxicity with failure of the blood count to recover by day 42 (six patients), patient refusal or death from an unknown cause (one each). Fewer patients receiving carboplatin and chlorambucil completed the treatment: in 15 cases this was due to progression, in 15 due to haematological toxicity and in one case each due to refusal or death from an unknown cause. The median total dose of carboplatin in Arm A was 2,400 mg $m^{-2}$ (minimum 400, 25 percentile 2,000, 75 percentile 2,800, maximum 2,900): and in Arm B 1,725 mg $m^{-2}$ (minimum 300, 25 percentile 900, 75 percentile 1,988, maximum 2,175).

Provision was made in the protocol for dose escalation in each course according to the nadir white blood cell and platelet counts in the preceding chemotherapy cycle. The dose of carboplatin could be escalated for at least one course in approximately 50% of patients. There was no substantial difference in the two arms in the patterns of dose reductions escalations or delays (Table III). Dose reductions were made in 52 cycles, and just over one quarter of patients had at least one dose reduction. A total of 62 cycles were delayed, 87% of them because of haematological toxicity (Table III).

### *Survival*

Figure 1 shows that there is no statistically significant difference in the PFS between the two arms. The median progression free survival for Arm A was 45 weeks and for Arm B was 61 weeks ($P = 0.830$). The relative progression rate is 0.96 (95% confidence interval 0.67–1.39) and these results are consistent with quite large treatment differences both in favour of and against the combination arm. Figure 2 shows survival according to the size of residual disease, again there was little difference between the two treatments (Figure 2). A variety of pre-treatment variables were examined to determine if they had any individual association with PFS: the results for the whole patient group are shown in Table IV. Only size of residual disease ($P = 0.0002$), performance status ($P = 0.004$) and FIGO stage ($P = 0.004$) showed any significant association with PFS. A multivariate Cox regression analysis showed that prognosis was mainly determined by performance status (0/1 *vs* 2) and size of residual disease; once these were allowed for, FIGO stage did not affect the outcome.

**Table I** Patient characteristics and treatment

| Characteristics | Carboplatin alone | Carboplatin chlorambucil | P-value |
|---|---|---|---|
| *Eligible for study* | $n = 81$ | $n = 71$ | |
| Age (yr) median | 58 | 55 | 0.142 |
| range | 29–75 | 21–74 | |
| *ECOG performance status* | | | |
| 0 | 42.0% (34) | 33.8% (24) | 0.570 |
| 1 | 43.2% (35) | 47.9% (34) | |
| 2 | 14.8% (12) | 18.1% (13) | |
| *FIGO stage* III | 79.0% (64) | 76.1% (54) | 0.663 |
| IV | 21.0% (17) | 23.9% (17) | |
| *Surgical procedure* | | | |
| BSO + h + o[a] | 18.5% (15) | 25.4% (18) | 0.066 |
| BSO + o[b] | 12.4% (10) | 25.4% (18) | |
| Lesser operations | 46.9% (38) | 36.3% (26) | |
| Biopsy alone | 22.2% (18) | 12.7% (9) | |
| *Residual tumour* | | | |
| <2 cm | 38.3% (31) | 32.4% (23) | 0.450 |
| ⩾2 cm | 61.7% (50) | 67.6% (48) | |
| *Histological type* | | | |
| Serous | 80.3% (65) | 64.8% (46) | 0.105 |
| Mucinous | 8.6% (7) | 12.7% (9) | |
| Clear cell | 6.2% (5) | 7.0% (5) | |
| Endometriod[c] | 1.2% (1) | 8.5% (6) | |
| Other[c] | 3.7% (3) | 7.0% (5) | |
| *Differentiation* | | | |
| Well[c] | 8.6% (7) | 2.8% (2) | 0.371 |
| Moderate[c] | 27.2% (22) | 22.5% (16) | |
| Poor | 54.3% (44) | 62.0% (44) | |
| Unknown | 9.9% (8) | 12.7% (9) | |
| *Centre* | | | |
| Aberdeen | 19.8% (16) | 18.3% (13) | 0.150 |
| Belfast | 17.3% (14) | 9.9% (7) | |
| Dundee | 21.0% (17) | 12.7% (9) | |
| Glasgow | 42.0% (34) | 59.2% (42) | |

[a]Bilateral salpingo-oophorectomy, total hysterectomy and omentectomy. [b]Bilateral salpingo-oophorectomy and omentectomy (in a proportion of these women the uterus had been removed at an earlier date for other indications). [c]These categories combined for calculating the *P*-values.

**Table II** Toxicity of treatment (worst toxicity recorded)

| | Carboplatin alone | Carboplatin and chlorambucil |
|---|---|---|
| White cell count[a] | | |
| 0 | 8.2% (6)[c] | 9.5% (6) |
| 1 | 31.5% (23) | 11.1% (7) |
| 2 | 46.6% (34) | 47.6% (30) |
| 3[b] | 13.7% (10) | 28.6% (18) |
| 4[b] | 0.0% (0) | 3.2% (2) |
| | | $P = 0.010$ |
| Platelet count[a] | | |
| 0 | 27.8% (20) | 37.1% (23) |
| 1 | 16.7% (12) | 11.3% (7) |
| 2 | 20.8% (15) | 27.4% (17) |
| 3 | 13.9% (10) | 14.5% (9) |
| 4 | 20.8% (15) | 9.7% (6) |
| | | $P = 0.304$ |
| Nausea and vomiting | | |
| 0 | 6.2% (5) | 5.6% (4) |
| 1 | 8.6% (7) | 8.5% (6) |
| 2 | 31.2% (26) | 40.9% (29) |
| 3 | 44.4% (36) | 39.4% (28) |
| 4 | 8.6% (7) | 5.6% (4) |
| | | $P = 0.823$ |

[a]Information on blood counts is only used for patients who had these recorded on all courses, for patients receiving ⩽ four courses; on at least four courses, for patients receiving ⩾ five courses. [b]These categories combined for calculating the *P*-value. [c]Number of patients.

**Table III** Patterns of dose escalations, reductions and treatment delays

| | Carboplatin alone (81)[a] | Carboplatin and chlorambucil (71)[a] | P-value |
|---|---|---|---|
| Dose escalations | | | |
| Escalated | 56.8% (46) | 47.9% (34) | 0.273 |
| Never escalated | 43.2% (35) | 52.1% (37) | |
| Dose reductions | | | |
| Reduced | 28.4% (23) | 26.8% (19) | 0.822 |
| Never reduced | 71.6% (58) | 73.2% (52) | |
| Dose delays | | | |
| Delayed | 29.6% (24) | 40.9% (29) | 0.148 |
| Never delayed | 70.4% (57) | 59.1% (42) | |

[a]Number of patients.

**Figure 1** Progression-free survival according to treatment arm.

| | No. at risk | No. progressed |
|---|---|---|
| ▲ JM8 + chlorambucil | 71 | 62 |
| ■ JM8 alone | 81 | 70 |

An analysis of progression free survival in terms of average haematological toxicity experienced by the patient was conducted. Actual nadir WBC and platelet counts for each cycle were used to calculate the overall average nadir values for each patient. In this analysis patients were first stratified according to the number of courses of chemotherapy they received; the intention of this was to remove any possible confusion of the comparison as a result of patients in the different haematological toxicity categories having received a different number of courses of chemotherapy and therefore having had different opportunites to achieve adequate myelosuppression. The analysis was then repeated adjusting for the two previously identified major prognostic-factors (performance status and extent of residual disease) by including these as co-variates in the proportional hazards model (Table V).

Patients whose average level of leucopenia did not exceed grade 0 had a worse outcome than those achieving higher grades (see Figure 3). A similar observation is seen with thrombocytopenia, but this does not achieve statistical significance. When leucopenia and thrombocytopenia are considered together in a multivariate analysis, the effect of thrombocytopenia is diminished, suggesting that leucopenia is the more important prognostic factor. Almost identical results are obtained if analysis is restricted to those patients who received at least three cycles.

The median disease-free survival in those with a clinical complete remission, i.e. patients with clinically measurable disease at the start of treatment, was 74 weeks (95% C.I.

**Figure 2** Progression-free survival in relation to the largest cross-sectional tumour diameter before initiation of chemotherapy. □, residual disease ⩾2 cm, carboplatin alone. Median PFS 41 weeks; 95% C.I. 28–48 weeks; △, residual disease ⩾2 cm, carboplatin and chlorambucil. Median PFS 33 weeks, 95% C.I. 23–65 weeks; ■, residual disease <2 cm, carboplatin alone. Median PFS 61 weeks, 95% C.I. 41–106 weeks; ▲, residual disease <2 cm, carboplatin and chlorambucil. Median PFS 92 weeks; 95% C.I. 61–? weeks. Relative progression rate (carboplatin and chlorambucil *vs* carboplatin alone) = 0.96 ($P = 0.830$). 95% C.I. for relative progression rate = 0.67–1.39.

**Table IV** Univariate analysis of association between pretreatment variables and progression-free survival

| *Pretreatment characteristics* | *Log-rank P-value* |
|---|---|
| Extent of residual disease (<2 cm *vs* ⩾2 cm) | 0.0002 |
| ECOG performance status (0/1 *vs* 2) | 0.004 |
| FIGO Stage (III *vs* IV) | 0.004 |
| Differentiation (well *vs* moderate *vs* poor) | 0.110 |
| Histological type (serous *vs* mucinous vs clear cell *vs* other) | 0.756 |
| Age (<50 *vs* ⩾50 years) | 0.421 |

**Table V** Association between average haematological toxicity (WHO grade) and progression-free survival

| | *WHO grade* | *No. of patients* | *P-value*[b] | *P-value*[c] |
|---|---|---|---|---|
| White cell count[a] | 0 *vs* 1/2/3 | 40 *vs* 96 | <0.0001 | <0.0001 |
| Platelets[a] | 0 *vs* 1/2/3 | 109 *vs* 25 | 0.056 | 0.052 |

[a]Information on blood counts is only used for patients who had these recorded: on all courses, for patients receiving ⩽four courses; on at least four courses, for patients receiving ⩾five courses. [b]The *P*-value obtained after stratifying for the number of cycles received. [c]This *P*-value is obtained after stratifying for the number of cycles received *and* including performance status and extent of residual disease in the proportional hazards model.

35–101 weeks). The median disease-free survival in the seven patients with pathological complete remission was 98 weeks.

The overall survival in the two groups was similar ($P = 0.552$) with the same median survival at 75 weeks as shown in Figure 4. Again the wide 95% confidence intervals of 0.77 to 1.64 for relative death rate indicate that quite large real survival differences between the treatment arms cannot be excluded. The amount of residual disease present before chemotherapy again influenced the long-term outcome as shown in Figure 5.

**Figure 3** Progression-free survival in relation to mean recorded white cell count nadir (WHO grade). ★, Grade 2/3; ▲, Grade 1 ■, Grade 0.

**Figure 4** Overall survival according to treatment arm.

| | No. at risk | No. death |
|---|---|---|
| ▲ JM8 + chlorambucil | 71 | 57 |
| ■ JM8 alone | 81 | 63 |

Tumour progression was the cause of 104 of the 120 patients who have died, eight died of unrelated causes and in seven the cause of death is unknown. These seven patients died at home or were lost to follow-up. There was one treatment related death in a patient who was conservatively managed in a peripheral hospital for intestinal perforation and haemorrhage occurring during the nadir of her first course whn the platelet count was $20 \times 10^9 l^{-1}$.

### Discussion

Carboplatin was introduced in Great Britain in April 1986 as an alternative to cisplatin with the objective of offering equal efficacy with less toxicity (Calvert *et al.*, 1982). To date the sample sizes in the studies comparing carboplatin and cis-

**Figure 5** Overall survival in relation to the largest cross-sectional tumour diameter prior to chemotherapy. □, Residual disease ⩾2 cm, carboplatin alone. Median survival 59 weeks; 95% C.I. 51–93 weeks; △, Residual disease⩾2 cm, carboplatin and chlorambucil. Median 51 weeks, 95% C.I. 37–72 weeks; ■, residual disease<2 cm, carboplatin alone. Median 107 weeks, 95% C.I. 75–193 weeks; ▲, residual disease<2 cm, carboplatin and chlorambucil. Median survival 151 weeks; 95% C.I. 94–? weeks. Relative death rate (carboplatin and chlorambucil vs carboplatin alone) = 1.12 ($P$ = 0.552). 95% C.I. for relative death rate = 0.77–1.64.

platin either as single agent or in combination are too small to show a definitive equivalence or otherwise between the two agents (Alberts et al., 1989; Edmonson et al., 1989; Ten Bokkel Huinink et al., 1987). Overviews of several randomised trials, which use a meta-analysis technique, can help to address the issue of limited numbers. Such a study has recently been conducted in ovarian cancer (Advanced Ovarian Cancer Trialists Group, 1991), and it points to the importance of long-term follow-up in comparative trials involving carboplatin.

Since randomised studies indicate that, at least in terms of response, the combination of an alkylating agent with cisplatin is superior to cisplatin alone (GICOG, 1987), a key issue for the future use of carboplatin will be its activity when combined with an alkylating agent. Our own pilot sudy had shown that a combination of carboplatin 300 mg m$^{-2}$ with chlorambucil 10 mg d × 7 days was well tolerated, and although dose reduction was sometimes required, dose escalation was possible in some patients (Harding et al., 1988).

In the randomised study reported here the dose for each cycle was based on the nadir blood count in the preceding cycle and both escalation and reduction were possible. Provision was made for only one escalation step. Further escalation of carboplatin would have been possible in some patients, as indicated by the fact that 31% of patients assessable for haematological toxicity only ever had mild (Grade 1 or less) myelosuppression. The interpatient variation in handling of carboplatin was idiosyncratic and did not correlate with age or disease stage (data not shown). No correlation was seen in the study reported here between received dose intensity for carboplatin and response, progression-free survival or overall survival.

We have observed a clear association between leucopenia and progression-free survival, and this is independent of other prognostic factors, i.e. performance status and extent of residual disease. While it is conceivable that patients prone to leucopenia have an inherently better prognosis, intuitively it is logical to suggest that interpatient variations in drug handling may affect outcome. A recent analysis of 767 patients treated with carboplatin for ovarian cancer has confirmed the relationship between tumour response and individual patient pharmacokinetics, i.e. area under the curve (Egorin et al., 1991). For these reasons it would be appropriate in patients receiving carboplatin alone or in combination to adjust the dose as necessary to achieve adequate myelosuppression (at least Grade 1) with each course of treatment. Adjustments of dose based on the criteria used in this study are simple to operate and easy to apply. Several other formulae are available for carboplatin alone (Egorin et al., 1985; Fish et al., 1987) or in combination (Calvert et al., 1989; Belani et al., 1989). We suggest that the individual variation in carboplatin handling can best be accommodated by determining the initial dose of carboplatin used alone or in combination on the basis of renal function (Calvert et al., 1989) and adjusting subsequent doses according to the nadir blood count.

One other study in ovarian cancer has shown that myelosuppression during chemotherapy confers an improved survival probability (GG.COSA, 1986). In that study which compared combination vs sequential therapy with chlorambucil and cisplatin, myelosuppression (defined as white cell count <2.5 × 10$^{9}$ l$^{-1}$ and/or platelets <100 × 10$^{9}$ l$^{-1}$) acted as an independent variable in the Cox multivariate analysis with a $P$ value of <0.001.

The dose of carboplatin used in either arm of our study is in line with that used in the other quoted studies. Mangioni and colleagues incorporated provision for dose escalation based on the blood count on the day of treatment, to a maximum of 500 mg m$^{-2}$ carboplatin by 50 mg m$^{-2}$ steps (Mangioni et al., 1989). In that study 38% patients had a dose escalation and 32% reduction. In our study, where the number of dose adjustments was similar in both arms and where the nadir blood count was used to guide the dose, 50% patients had at least one dose escalation and 25% a dose reduction.

In conclusion, we have compared carboplatin alone with a combination of carboplatin and chlorambucil in a randomised study in patients with Stage III and IV ovarian cancer. We found no statistically significant difference in the progression-free survival or overall survival between the two treatments. It therefore seems unlikely that there is any major advantage (i.e. median survival difference of 30% or more) to adding an alkylating agent, at least chlorambucil, to single agent carboplatin for the management of this disease. The amount of residual disease at the start of chemotherapy and the performance status were the most important predictors for progression-free survival. Although the median overall survival seen in both arms in this study of 75 weeks, is less than that which can be achieved with cisplatin-based combination therapy, proper comparisons can only be made on prospective randomised studies with long-term follow-up.

The authors thank their colleagues in Scotland for referring patients for this study, Dr Nanette Gordon and Mrs Laura McNulty for their help with data management, the Cancer Research Campaign for support of the West of Scotland Clinical Trials Office, Bristol Myers for supplying some of the carboplatin used in the study and Marie Anne van Halem and Marion McLeod for their secretarial assistance.

### References

ADVANCED OVARIAN CANCER TRIALISTS GROUP (1991). Chemotherapy in advanced ovarian cancer: an overview of randomised clinical trials. *Br. Med. J.*, **303**, 884–893.

ALBERTS, D., GREEN, S., HANNIGAN, E. & 7 others (1989). Improved efficacy of carboplatin (carboP)/cyclophosphamide (CPA) vs cisplatin (cisP)/CPA: preliminary report of a phase III, randomized trial in stages III-IV, suboptimal ovarian cancer (OV CA). *Proc. Am. Soc. Clin. Oncol.*, **8**, 151 (Abstract).

BELANI, C.P., EGORIN, M.J., ABRAMS, J.S. & 4 others (1989). A novel pharmacodynamically based approach to dose optimization of carboplatin when used in combination with etoposide. *J. Clin. Oncol.*, **7**, 1896.

CALVERT, A.H., NEWELL, D.R., GUMBRELL, L.A. & 7 others (1989). Carboplatin dosage: prospective evaluation of a simple formula based on renal function. *J. Clin. Oncol.*, **7**, 1748.

DEMBO, A.J. (1986). Controversy over combination chemotherapy in advanced ovarian cancer: what we learn from reports of matured data. *J. Clin. Oncol.*, **4**, 1573.

EGORIN, M., JODRELL, D., CANETTA, R. & 6 others (1991). Tumour response and toxicity in ovarian cancer correlates with carboplatin area under the curve. *Proc. Amer. Soc. Clin. Onc.*, **10**, 184.

EDMONSON, J.H., MCCORMACK, G.M., WIEAND, H.S. & 11 others (1989). Cyclophosphamide-cisplatin versus cyclophosphamide-carboplatin in stage III–IV ovarian carcinoma: a comparison of equally myelosuppressive regimens. *J. Natl Cancer Inst.*, **81**, 1500.

EGORIN, M.J., VAN ECHO, D.A., OLMAN, E.A., WHITACRE, M.Y., FORREST, A. & AISNER, J. (1985). Prospective validation of a pharmacologically based dosing scheme for the cis-diamminedichloro- platinum (II) analogue diamminecyclobutanedicarboxylatoplatinum. *Cancer Res.*, **45**, 6502.

EVANS, B.D., RAJU, K.S., CALVERT, A.H., HARLAND, S.J. & WILTSHAW, E. (1983). Phase II study of JM8, a new platinum analog, in advanced ovarian carcinoma. *Cancer Treat. Rep.*, **67**, 997.

FISH, R.G., ADAMS, M., SHELLY, M.D. & 4 others (1987). Validity of a dosing scheme for carboplatin. *Proc. Am. Soc. Clin. Oncol.*, **6**, 21.

GRUPPO INTEREGIONALE COPPERATIVO ONCOLOGICO GINECOLOGICA (1987). Randomised comparison of cisplatin with cyclophosphamide/cisplatin and with cyclophosphamide/doxorubicin/cisplatin in advanced ovarian cancer. *Lancet*, **ii**, 353.

GYNAECOLOGICAL GROUP, CLINICAL ONCOLOGICAL SOCIETY OF AUSTRALIA, Sydney Branch, Ludwig Institute for Cancer Research (1986). Chemotherapy of advanced ovarian adenocarcinoma: a randomized comparison of combination versus sequential therapy using chlorambucil and cisplatin. *Gynecol. Oncol.*, **23**, 1.

HARDING, M., KENNEDY, R., MILL, L. & 5 others (1988). A pilot study of carboplatin (JM8, CBDCA) and chlorambucil in combination for advanced ovarian cancer. *Br. J. Cancer*, **58**, 640.

HRYNUIK, W.M. (1987). Average relative dose intensity and the impact on design of clinical trials. *Semin. Oncol.*, **14**, 65.

MANGIONI, C., BOLIS, G., PECORELLI, S. & 10 others (1989). Randomized trial in advanced ovarian cancer comparing cisplatin and carboplatin. *J. Natl Cancer Inst.*, **81**, 1464.

OZOLS, R.F. & YOUNG, R.L. (1984). Chemotherapy of ovarian cancer. *Semin. Oncol.*, **11**, 251.

OZOLS, R.F. (1985). The case for combination chemotherapy in the treatment of advanced ovarian cancer. *J. Clin. Oncol.*, **3**, 1445.

OZOLS, R.F., OSTCHEGA, Y., MYERS, C.E. & YOUNG, R.C. (1985). High dose cisplatin in hypertonic saline in refractory ovarian cancer. *J. Clin. Oncol.*, **3**, 1246.

SEROV, S.F., SCULLY, R.E. & SOBIN, L.H. (1973). *International histological classification of tumours No. 9. Histological typing of ovarian tumours.* WHO: Geneva, p. 10–21.

TEN BOKKEL HUININK, W.W., VAN DER BURG, M.E.L., VAN OOSTEROM, A.T., DALESIO, O., ROTMENSZ, N. & VERMORKEN, J.B. (1987). Carboplatin replacing cisplatin in combination chemotherapy for ovarian cancer. A large scale randomized phase III trial of the Gynecological Cancer Cooperative Group of the EORTC. *Proc. Am. Soc. Clin. Oncol.*, **6**, 118 (Abstract).

WHO HANDBOOK FOR REPORTING RESULTS OF CANCER TREATMENT (1979). *WHO Offset Publication, number 48*, WHO: Geneva.

WILLIAMS, C.J., MEAD, G.M., MACBETH, F.R. & 8 others (1985). Cisplatin combination chemotherapy versus chlorambucil in advanced ovarian carcinoma: mature results of a randomized trial. *J. Clin. Oncol.*, **3**, 1455.

YOUNG, R.C., HUBBARD, S.P. & DE VITA, V.T. (1974). The chemotherapy of ovarian cancer. *Cancer Treat. Rev.*, **1**, 99.